# Human Factors Usability and Validation Studies of a Glucagon Autoinjector in a Simulated Severe Hypoglycemia Rescue Situation

**DOI:** 10.1089/dia.2019.0148

**Published:** 2019-08-19

**Authors:** Virginia Valentine, Brett Newswanger, Steve Prestrelski, Anthony D. Andre, Mark Garibaldi

**Affiliations:** ^1^ Clinica La Esperanza, Albuquerque, New Mexico.; ^2^ Xeris Pharmaceuticals, Inc., Chicago, Illinois.; ^3^ Interface Analysis Associates, Saratoga, California.

**Keywords:** Hypoglycemia, Glucagon, Human factors, Usability, Validation

## Abstract

***Background:*** A room-temperature stable, soluble liquid glucagon formulation loaded into a prefilled, single-use, two-step autoinjector is under development for severe hypoglycemia rescue. We report a human factors validation program evaluating the glucagon autoinjector (GAI) (Gvoke HypoPen™; Xeris Pharmaceuticals, Inc., Chicago, IL) versus marketed glucagon emergency kits (GEKs) for managing severe hypoglycemia.

***Methods:*** A simulated-use human factors usability study was conducted with the GAI versus marketed GEKs in 16 participants, including adult caregivers and first responders, experienced with glucagon administration. A summative human factors validation study of the GAI was conducted with 75 volunteers. Participants were (1) trained on the device and procedure or (2) given time to individually read the instructions and familiarize themselves with the device. Participants returned a week later to perform an unaided rescue attempt that simulated rescue of patients with diabetes suffering a hypoglycemia emergency. Participant actions were recorded for critical rescue tasks and use errors.

***Results:*** In the usability study, 88% (14) successfully administered a rescue injection using the GAI versus 31% (5) using GEKs (*P* < 0.05). Mean total rescue time of use was 47.9 s with the GAI versus 109.0 s with GEKs (*P* < 0.05). In the validation study, 98.7% successfully administered the rescue injection using the GAI. Overall, there were no patterns of differences between trained versus untrained participants, between caregivers versus first responders or between adults versus adolescents.

***Conclusion:*** The GAI and instructional materials can be correctly, safely, and effectively used by intended user, which support continued development of the GAI as an alternative to GEKs.

## Introduction

Severe hypoglycemia is a condition where a person with diabetes experiences a low blood glucose level that leads to clinical symptoms, including serious cognitive impairment, and requires external assistance to recover. Severe hypoglycemia is a major concern of patients with diabetes and their caregivers, and when undertreated, episodes can lead to coma, seizures, and occasionally death.^1–4^ Hypoglycemic episodes among patients with both type 1 and type 2 diabetes are associated with an economic and emotional burden,^5–7^ with a substantial proportion of patients expressing fears or worry about the occurrence of hypoglycemia.^8^ Caregivers of persons with diabetes may also experience distress and feel unable to adequately undertake the administration of glucagon, especially during an emergency situation.^9^

Based on American Diabetes Association (ADA) clinical practice guidelines, glucagon should be prescribed for all individuals at increased risk of clinically significant hypoglycemia defined as blood glucose <54 mg/dL (3.0 mmol/L).^10^ The currently approved glucagon emergency kits (GEKs) for severe hypoglycemia rescue contain lyophilized powder formulations of glucagon in a vial that require manual reconstitution with an aqueous diluent in a syringe format at time of use due to the instability of glucagon peptide in solution. Once reconstituted, glucagon is subject to unfolding at hydrophobic peptide interfaces, which leads to the formation of non-native β-sheets and ultimately the rapid formation of toxic fibrils.^11^

While a lyophilized powder prevents degradation of glucagon, the vial and syringe formats are difficult to administer and are not well accepted by users.^12^ This multistep drug preparation in an emergency setting, often performed by inadequately trained caregivers and personnel, frequently results in errors where the patient does not receive a full dose of glucagon.^12^

The poor usability of GEKs may also be reflective in the low uptake of the drug in the diabetes population, where approximately half of persons with diabetes prescribed a GEK decide to continue fulfilling their renewal prescription.^13^ Furthermore, the overall utilization of glucagon reflects less than one-fifth of the addressable population—for example, those who take insulin and are at risk of clinically significant hypoglycemia. Experts conclude that training on glucagon administration requires “hands on” practice and follow-up assessment of skills.^9^

Intuitive and easier-to-use glucagon preparations^14^ improve the usability, may reduce the training burden, and may improve the overall utilization of glucagon in hypoglycemia emergencies. Human factor studies of GEKs, where caregivers or emergency personnel are asked to administer glucagon in a simulated emergency situation, demonstrate that only a small minority of users are able to prepare and properly administer the full dose of drug.^15,16^

Hence, the optimal scenario during a diabetes emergency is the delivery of glucagon in a ready-to-use format that is user-centric, intuitive, reliable, and enables complete dose delivery. Thus, a need exists for an improved device for hypoglycemic rescue that provides ease-of-use for all potential users as well as reliable delivery of a complete dose of glucagon.

**FIG. 1.**
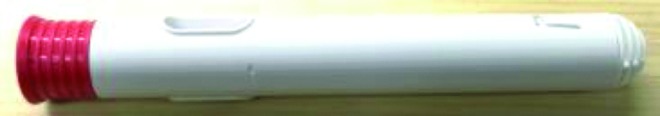
Glucagon autoinjector (Gvoke HypoPen™, Xeris Pharmaceuticals, Inc., Chicago, IL).

A novel, soluble, liquid glucagon formulation that is stable for at least 2 years at room temperature is in development for severe hypoglycemia rescue (Fig. 1).^17^ This formulation is accomplished through a nonaqueous platform technology that inhibits glucagon fibrillation and provides enhanced stability and full portability. The stable liquid glucagon is loaded in a prefilled, ready-to-use autoinjector and has been successfully tested across multiple Phase 3 studies during conditions of insulin-induced severe hypoglycemia.^18,19^ The autoinjector offers the potential for easy, rapid injection of a full dose of glucagon during emergencies and further reduces fear and anxiety for both the patient and caregiver.

Comparative usability and validation studies were undertaken to evaluate the glucagon autoinjector (GAI) (Gvoke HypoPen™; Xeris Pharmaceuticals, Inc., Chicago, IL) versus currently marketed GEKs. The objectives of the comparative usability study were to (1) evaluate the intuitiveness, ease of use, and acceptability of the GAI; (2) evaluate the readability and effectiveness of the instructions for use (IFU) and quick-use Label Guide (printed directly on the secondary device packaging) to identify any necessary changes and to further optimize before validation; (3) evaluate how the GAI compared to GEKs (with vial and syringe) for ease of use and preference; and (4) finalize the device, IFU, and test method in preparation for the validation study.

The objectives of the validation study were to (1) validate whether GAI procedure and instructional materials (Dose Label, Packaging, IFU, and Label Guide) could be correctly, safely, and effectively used by the intended user populations (Adult and Adolescent Family/Caregivers and First Responders) and (2) determine if specific aspects of the GAI Label Guide, and/or IFU led to any patterns of high-risk use errors when used by the intended populations.

## Methods

### Comparative study

The comparative usability study was conducted with a group of 16 participants consisting of 8 caregivers of patients with diabetes or first responders who were experienced with using GEKs, and 8 adults with no relationship to a diabetes patient and naive to GEKs. Half of each participant group received training and the other half received no training.

Before each participant arrived, the test room was set up to simulate a real-life atmosphere to create the conditions a typical user would experience when using the device being tested. For this study, the room was set up with a mannequin in the middle of the room on the floor, to represent a patient with diabetes experiencing a hypoglycemic emergency. A tote bag containing the glucagon rescue device (kit or autoinjector) was placed on the floor next to the mannequin. A sound file was utilized to play sounds that represent a normal ambient environment: in this study, the recording simulated a restaurant setting (background conversations, silverware clanking, and so on). A table and chairs to the side of the room were used during training and the postinteraction interview.

Trained participants received a short, representative training from the moderator on how to prepare and administer a glucagon injection. Glucagon-experienced participants only received training on the GAI, while the glucagon-naive participants received training on both the GAI and a GEK. Training included an overview of the device and a verbal walk-through of the procedure. Untrained participants did not receive any form of training from the moderator.

**FIG. 2.**
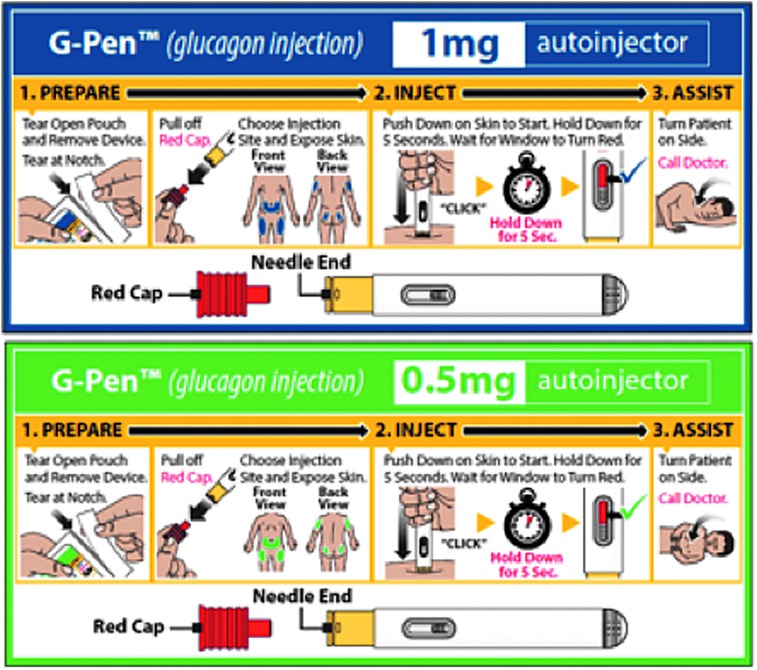
Label Guide and pouch for 1 mg adult dose (top) and 0.5 mg pediatric dose (bottom).

**FIG. 3.**
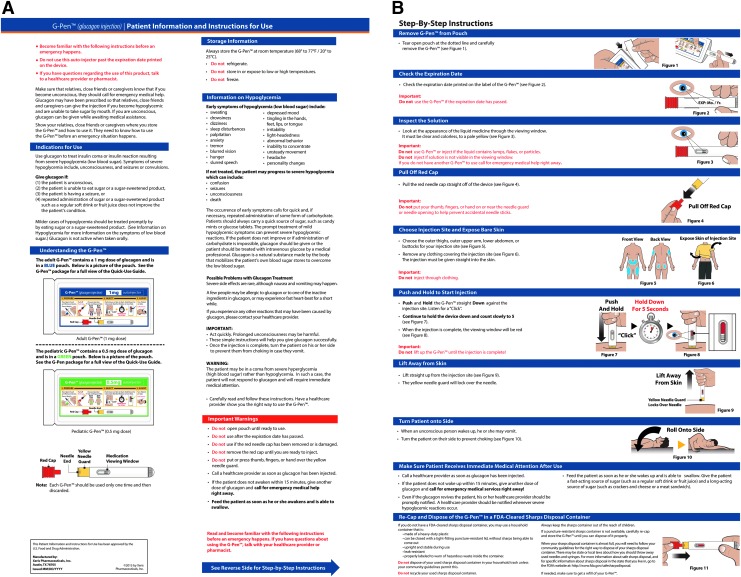
Instructions for use.

All participants performed one unaided rescue injection with a GEK and one unaided rescue injection with the GAI (counterbalanced order). After each rescue attempt, participants provided postinteraction feedback on their experience. After both rescue injections were performed, participants compared the devices, provided feedback on the Label Guide (Fig. 2) and IFU (Fig. 3), and indicated their device preferences.

During each session, study personnel observed the performance and behaviors of each participant. Success, failure, errors, confusion, and other indices of a poor interaction that could result in incorrect use of the device were recorded. Participants also were interviewed to provide a subjective narrative of their experience and opinions of the device and IFU. A successful injection was defined as any injection where the participant correctly prepared and injected the full drug dose with the given rescue kit.

### Validation study

The summative human factors validation study consisted of two sessions. In the first session, participants either received training or underwent self-familiarization with glucagon administration procedures (Table 1). In the second session, participants administered the GAI under simulated emergency conditions with assessment of their skills and experiences.

**Table 1.** Study Design Overview (75 Total Unaided Rescue Attempts)

	*User group 1: first responders*	*User group 2: experienced caregivers*	*User group 3: naive caregivers*		
Number of participants	15	15	15	15	15
Age group	Adult	Adult	Adult	Adult	Adolescent
Glucagon kit experience	Yes	Yes	No	No	No
Training	Trained	Untrained (self-familiarized)	Trained	Untrained (self-familiarized)	Trained
Injection site	Selected by participant	Selected by participant	Selected by participant	Selected by participant	Selected by participant
Rescue attempts	1	1	1	1	1

The study included 75 participants divided into 3 user groups. Group 1, First Responders, consisted of 15 individuals who represented the types of people who would likely be available to respond to a patient with diabetes experiencing a hypoglycemic emergency, including emergency response personnel, schoolteachers, and school nurses. All the First Responders were trained on how to use the GAI during their first session consistent with current real-world training practices. Group 2, Experienced Caregivers, consisted of 15 caregivers who had experience with one of the GEKs currently on the market. Caregiver participants included close family members, friends, coworkers, or people who would likely be with a patient with diabetes when a hypoglycemic event occurs and would know to attempt a glucagon rescue.

All the Experienced Caregivers were untrained (self-familiarized) because it is possible that Experienced Caregivers may not be given training when a diabetes patient is prescribed a new drug. Instead diabetes patients would likely be sent home with the glucagon device and IFU, and it would be up to the caregiver to become familiar with how to use the device before administering an injection in an emergency setting. Group 3, Naive Caregivers, consisted of 30 Adult Caregivers and 15 Adolescent (12–17 years old) Caregivers who had no experience with glucagon in the past. Similar to Group 2, caregiver participants in this group included close family members, friends, coworkers, or people who would likely be with a patient with diabetes when a hypoglycemic event occurred and would know how to attempt a glucagon rescue.

To be consistent with real-world training practices, Naive Adult Caregivers were separated into 2 training conditions that included 15 trained and 15 untrained (self-familiarized) participants. All Adolescent Caregivers were trained. Sample sizes of at least 15 per user group met the minimum number of participants required for a human factors validation study^19^ across the spectrum of end-user demographics.

#### Learning conditions

All First Responders (*n* = 15) received training before performing an unsupervised glucagon rescue attempt. In addition, half of the Naive Caregivers (*n* = 15) and all Naive Adolescent Caregivers (*n* = 15) received training. To test the worst-case scenario, all Experienced Caregivers (*n* = 15) and the remaining Naive Caregivers (*n* = 15) received no training before performing an unaided rescue attempt.

Trained participants received a very brief but representative training, which included an introduction to the device and drug, a verbal walk through, and time to read the instructions. They were not allowed to perform a practice injection with the device.

Untrained participants did not receive any training, but instead were allowed time to familiarize themselves with the device and IFU as needed. As in the real-world, these participants did not receive any guidance or instruction about what to review or how to familiarize themselves with the device.

To simulate a realistic decay in knowledge, training (for trained users) and self-familiarization (for untrained users) took place during the first study session, which occurred 1 week before the second session, where participants took part in an unaided rescue attempt.

The recommended injection sites for GAI were the subcutaneous tissue at the upper arms, upper buttocks, abdomen, and thigh. All participants were observed as to which injection site they selected to administer the injection into the mannequin. None of the participants was instructed or assigned an injection site.

#### Test room setup

Before each participant arrived, the test room was set up to simulate a real-world atmosphere and to create conditions which the user might experience when using the device being tested. For this study, the room was set up with a mannequin lying on the floor in the center of the room to represent a patient with diabetes experiencing a hypoglycemic emergency. A sound file continuously played atmospheric sounds that would occur in a rescue situation, including sirens, cars honking, and people speaking in the background. The glucagon device in a pouch with Label Guide was provided in a tote bag next to the mannequin, simulating how most patients might carry the product. The complete IFU, reviewed the week before, was not available during the rescue attempt. Finally, a table and two chairs were positioned to the side of the room for use during training and postinteraction questions.

#### Human factor assessments

During each session, study staff observed the performance and behaviors of each participant. Success, failure, errors, confusion, and other indices of malinteraction that could result in incorrect use of the device were recorded (Table 2). Participants were also interviewed to provide a subjective narrative of their experience and opinions of the device, injection procedure, Label Guide, and IFU.

**Table 2.** Study Assessments for Validation Study

*Task*	*Study technique*	*Range of acceptable performance or verbal response*
Remove device from pouch	Task: Participants were observed regarding whether or not they open the pouch and remove the device.	Performance data: Must remove device from pouch.
		Subjective data: Verbal response to any difficulty with any aspect of the process.
Remove device cap	Task: Participants were observed regarding whether or not they remove the device cap.	Performance data: Must remove device cap.
		Subjective data: Verbal response to any difficulty with any aspect of the process.
Expose skin on injection site	Task: Participants were observed regarding whether or not they expose and inject into bare skin.	Performance data: Must expose bare skin of mannequin. Must identify the need to inject into bare skin.
	Knowledge probe: Participants were asked to identify the need to inject into bare skin.	Subjective data: Verbal response to any difficulty with any aspect of the process.
Activate injection	Task: Participants were observed regarding whether or not they activate the injection.	Performance data: Must press down to activate injection.
		Subjective data: Verbal response to any difficulty activating the injection and administering the full dose.
Administer full dose	Task: Participants were observed regarding whether or not they held the device down long enough to administer the full dose.	Performance data: Must hold the device down long enough to deliver the full dose.
		Observation: Does not spill clinically significant amount of drug.
		Subjective data: Verbal response to any difficulty administering the full dose and knowing when the injection was complete.

- **Performance Measures:** A successful injection was defined as any injection where the participant performed the correct procedure with the injection device and administered the full dose.
- **Behavioral Measures:** Behavioral measures included indices of excessive effort or frustration and verbal comments made by the participant during the study (when applicable).
- **Subjective Measures:** After performing an injection using the injection device, participants were asked to provide subjective feedback on various aspects of the procedure. Participants also provided subjective feedback related to the device, injection procedure, Label Guide, and IFU.

Successful validation was demonstrated by the absence of any pattern of preventable use failure or difficulties with the device, procedure, and instructional materials.

## Results

Baseline demographic characteristics for the Comparative and Validation studies are shown in Table 3.

**Table 3.** Baseline Demographics for Comparative and Validation Studies

	*Comparative study*		*Validation study*			
	*Naive (*n* = 8)*	*Experienced (*n* = 8)*	*First responders (*n* = 15)*	*Experienced caregivers (*n* = 15)*	*Naive caregivers (*n* = 30)*	*Naive adolescent caregivers (*n* = 15)*
Age, years^a^	35.5 ± 14.9	37.4 ± 11.5	38.0 ± 12.5	49.5 ± 8.4	40.3 ± 14.5	14.7 ± 0.5
Age range	19–58	20–55	24–64	35–69	18–67	12–17
Female, *n* (%)	4 (50)	5 (62)	7 (47)	10 (67)	15 (50)	6 (40)
Ethnicity, *n* (%)						
Asian	2 (25.0)	2 (25.0)	1 (6.7)	4 (27.0)	7 (23.0)	2 (13.0)
Caucasian	2 (25.0)	5 (62.5)	11 (73.2)	8 (53.0)	9 (30.0)	7 (47.0)
Hispanic	3 (37.5)	0	1 (6.7)	2 (13.0)	8 (27.0)	3 (20.0)
Pacific Islander	1 (12.5)	0	1 (6.7)	0	3 (10.0)	1 (7.0)
Other^b^	0	1 (12.5)	1 (6.7)	1 (7.0)	3 (10.0)	2 (13.0)
Education level, *n* (%)						
High school	4 (50.0)	1 (12.5)	1 (6.7)	0	6 (20.0)	NA
Associate degree	0	3 (37.5)	7 (46.6)	5 (33.3)	8 (27.0)	NA
Bachelor degree	3 (37.5)	2 (25.0)	4 (26.7)	5 (33.3)	15 (50.0)	NA
Master's degree	1 (12.5)	2 (25.0)	3 (20.0)	4 (26.7)	1 (3.0)	NA
Professional degree	0	0	0	1 (6.7)	0	NA

^a^ Mean ± standard deviation

^b^ Other = African American (4), East Indian (3), Persian (1)

NA, not applicable; all adolescent caregivers were in Grades 6 to 11

### Comparative study

Overall, 14 of 16 participants (88%) were able to successfully administer a rescue injection using the GAI compared with 5 of 16 participants (31%) with the GEK (chi-square test = 10.49, *P* < 0.05) (Table 4). The observed causes of failure for the GAI included the following: (1) could not remove device cap and (2) injected through clothing. The observed causes of failure for the GEK included the following: (1) bent needle, (2) injected through clothing, (3) injected diluent only, (4) did not fully reconstitute, and (5) did not inject entire volume. Furthermore, while some participants may have correctly followed the GEK reconstitution and injection procedure, it was observed that many vials still contained solution or powder. In fact, only 6/16 (38%) of the GEK vials were empty following the injection procedure. Two vials still contained powder, and on average, ∼0.5 mL remained in each of the eight vials that still contained liquid. This is significant as it is about half of the reconstituted volume of the 1 mL recommended, full adult dose of glucagon (concentration of 1 mg/mL).

**Table 4.** Results from Comparative Study

*Comparative performance measures*	*GAI*	*GEK*
Successful dose administrations	14/16 (88%)	5/16 (31%)
Administered with reduced efficacy	0/16 (0%)	4/16 (25%)
Failed administration	2/16 (13%)	7/16 (44%)
Error rate	3.6%	20.1%
Mean total rescue time (start at entry of room until delivery of dose)	47.9 s	109.0 s
Median total rescue time (start at entry of room until delivery of dose)	35.0 s	96.0 s

GAI, glucagon autoinjector; GEK, glucagon emergency kit.

It should be noted that in the context of glucagon human factors studies, injection through clothing was considered a failure. The GEK instructs users to inject on skin surfaces,^20^ and similarly the labeling of the GAI is for injection on skin surfaces only, to ensure proper injection depth and delivery of a full dose of glucagon. In this study, one failure with the GAI and three failures with the GEK were attributed to (or at least partially attributed to) injection through clothing.

Finally, a significantly lower step error rate of 3.6% was observed with the GAI versus 20.1% for the GEKs [*F*(1, 28) = 8.89, *P* < 0.05], and a significantly faster mean total rescue time of 47.9 s was observed with the GAI versus 109.0 s for the GEKs [*F*(1, 28) = 12.41, *P* < 0.05].

Having established that the GAI has significant benefits, both in rescue time and success, over the GEK, we next endeavored to more formally validate the GAI device, procedure and labeling per the Food and Drug Administration (FDA) guidance.^21^

### Validation study

Overall, 74 of 75 participants (98.7%) successfully administered the rescue injection using the GAI (Table 5). All participants (1) successfully removed the device from the pouch, (2) removed the cap from the device, (3) selected an appropriate injection site, (4) exposed the skin of the mannequin, and (5) activated the injection by pressing the device against the skin.

**Table 5.** Performance Measures–Rescue Injection Findings–Validation Study

	*Trained*			*Untrained*		
	*First responders (*n* = 15)*	*Naive adult caregivers (*n* = 15)*	*Naive adolescent caregivers (*n* = 15)*	*Naive adult caregivers (*n* = 15)*	*Experienced adult caregivers (*n* = 15)*	*Overall (*n* = 75)*
Failure to open and remove device from pouch	0	0	0	0	0	0
Failure to remove cap from device	0	0	0	0	0	0
Did not inject into one of the recommended injection sites (error)	0	0	0	0	0	0
Injection site used						
Outer thighs	0	0	0	1 (7%)	1 (7%)	2 (3%)
Outer upper arm	0	0	0	0	0	0
Lower abdomen	15 (100%)	15 (100%)	15 (100%)	13 (87%)	14 (93%)	72 (96%)
Buttocks	0	0	0	1 (7%)	0	1 (1%)
Did not expose skin on site (error)	0	0	0	0	0	0
Failure to activate injection	0	0	0	0	0	0
Failure to administer full dose	0	0	0	1 (7%)	0	1 (1%)
Failure to roll manikin onto side	0	0	0	0	0	0
Needle stick (error)	0	0	0	0	0	0
Refers to Label Guide	15 (100%)	13 (87%)	14 (93%)	14 (93%)	14 (93%)	70 (93%)
Instances of difficulty	0	0	0	0	0	0

Overall, there were no patterns of differences between trained versus untrained participants, between caregivers versus first responders or between adults versus adolescents. All participants stated that they did not have any difficulty with any aspect of the process and that they did not have any concerns about their ability to safely and effectively use the GAI to give an injection in an actual emergency situation. Several participants gave unsolicited comments that the injection process was extremely easy, and the Label Guide supported them through the rescue injection process.

One participant, an untrained Naive Adult Caregiver, failed to administer the full dose. The participant performed all preliminary steps of the rescue injection procedure correctly (removing the pen from the pouch, removing the red pen cap, identifying the injection site, exposing the injection site, and activating the pen injection) as well as concluding steps (rolling patient on side). However, the participant lifted the pen prematurely from the injection site, within 1.5 s of activating the pen, which resulted in the patient receiving a partial dose. The failure debrief revealed that the participant only used the Label Guide to identify the injection site and did not utilize the other steps displayed on the Label Guide. The participant highlighted that if she had read the Label Guide during the full process, she would have performed the injection procedure correctly. The participant was asked to perform a second unaided rescue injection with the GAI and demonstrated that she could perform every step of the process while reading the instructions on the Label Guide.

## Discussion

The burden of hypoglycemia is prevalent and impacts the lives of persons with diabetes and the communities around them. Hypoglycemia is common in patients using insulin analog regimens, and even nonsevere hypoglycemia is associated with clinically significant effects on patient well-being and functioning, patient and physician management of glycemic targets, and overall health care utilization.^22^ The benefits of achieving tight glycemic control (e.g., lower glycemic targets) in patients with both type 1 and type 2 diabetes are well known but are counterbalanced by the ongoing risk of symptomatic hypoglycemia.^9^ Symptomatic hypoglycemia in patients with diabetes remains a persistently under addressed issue. It is associated with frequent complications, especially in children, and is a significant deterrent that limits achieving normoglycemia.^23^

Both patients and caregivers need better education on treating hypoglycemia to alleviate fears and remove knowledge-based and physical barriers from administration of glucagon during an emergency.^9^ New and improved methods for the administration for glucagon, with better functional efficacy, are needed to help alleviate fears and encourage caregivers to administer appropriate glucagon therapy. During a hypoglycemia emergency, a person with diabetes should receive a full dose of glucagon in both a timely and reliable manner.

Previous simulation studies with GEKs have documented their limitations. Among 24 participants who were experienced with the use of GEKs, naive to GEKs, or bystanders, only 6% delivered a full dose of glucagon compared to 87% with an investigational easily reconstituted product, and administration time was markedly slower with the GEK.^15^ Another simulation study evaluated a needle-free glucagon nasal powder versus a marketed GEK in 16 caregivers as well as acquaintances.^16^ Only 12.5% of caregivers and none of the acquaintances delivered a full dose of glucagon from the GEK in this study.

Another simulation examined the technique for administering glucagon via GEK among 136 parents of children with diabetes.^24^ Difficulties with handling the administration, including opening the container and withdrawing the proper dose were observed in 69% of parents. Results from these simulation studies are consistent with the usability study reported here and highlight the need for alternative products for administering glucagon.

The current comparative usability study found that the GAI is a viable alternative to a currently marketed GEKs. Moreover, the performance results across experienced, naive, trained, and untrained participants showed the GAI to be safer, easier, and faster to use while leading to more successful simulated rescues. This was supported by a notable preference of the GAI over the GEKs by all participants. In addition, when compared to the GEK, all participants found the GAI to be easier to prepare, easier to inject, easier to remember, more convenient, safer, and faster to use. The IFU and Label Guide associated with the GAI performed well and were found to be clear and readable by participants.

In the formative study, there was one injection through clothing failure observed with the GAI compared with three failures with the GEK. In bench testing, injection with the GAI through various types of clothing has been performed with positive results (e.g., full dose delivery of glucagon). However, since there is wide variability in the thickness of clothing, which may affect the penetration depth of a subcutaneous needle, it was determined from a risk perspective that users should continue to follow current labeling to perform the injection on exposed skin surfaces. In the subsequent summative study, no users performed the injection through clothing. Thus, these results demonstrate that current labeling and recommended injection procedure can successfully mitigate injection errors.

Notwithstanding the positive results, there were two issues observed during rescue injections with the GAI related to removing the injector from the carrier tube and the removal of the autoinjector cap. While these issues did not impact the overall positive performance of the device, recommendations were made to mitigate these difficulties through enhancements to the instructional materials and the device packaging. For example, the device packaging changed from a cigar-like tube to an easy-tear pouch, so there was only one device cap for users to remove to avoid the confusion experienced in the formative study. Following the implementation of these recommendations, the device and instructional materials were ready for validation.

Based on results from the validation study, the GAI was safe, effective, and usable for the intended users and use contexts. The study validated that intended users could easily and correctly differentiate between the adult (1 mg) and pediatric (0.5 mg) doses via the labeling. The study also validated that intended users could successfully open the sealed foil pouch and remove the device without incident and successfully deploy the device and carry out the full rescue injection procedure in an emergency use context. Intended users could learn from, comprehend, and follow the Label Guide, and could learn from, comprehend, and recall instructions for the IFU, and translate these instructions into successful product interaction during the rescue attempt. Users found the Label Guide and IFU acceptable.

Further support for the viability of GAI is provided by results from Phase 3 studies of GAI versus a marketed GEK where the two product presentations showed similar efficacy for glucose peak concentration, time to peak concentration, and area under the concentration:time curve time 0–90 min.^18,19^

In summary, these results support the GAI as a viable alternative to currently marketed GEKs for rescue treatment of severe hypoglycemia in patients with diabetes. Compared to GEKs, the GAI has high functional efficacy because it can be successfully used by trained and untrained adolescent and adult caregivers to deliver a full rescue dose of glucagon both consistently and reliably. This usability advantage over existing GEKs will hopefully inspire more confidence in caregivers of patients with diabetes and translate to more widespread use of glucagon in treating severe hypoglycemia in the home or ambulatory setting. As such, the GAI could not only help save lives but also limit emergency department visits, and thus lower the burden of severe hypoglycemia on the health care system as a whole.
